# Role of energy metabolic deficits and oxidative stress in excitotoxic spinal motor neuron degeneration *in vivo*

**DOI:** 10.1042/AN20130046

**Published:** 2014-03-12

**Authors:** Luz Diana Santa-Cruz, Ricardo Tapia

**Affiliations:** *División de Neurociencias, Instituto de Fisiología Celular, Universidad Nacional Autónoma de México, 04510-México, D.F., México

**Keywords:** amyotrophic lateral sclerosis, antioxidant, mitochondrial energy substrate, motor neuron degeneration, oxidative stress, spinal cord, ALS, amyotrophic lateral sclerosis, AMPA, α-amino-3-hydroxy-5-methyl-4-isoxazole propionate, ChAT, choline acetyltransferase, GEE, GSH ethyl ester, MN, motor neuron, 3-NT, 3-nitrotyrosine, ROS, reactive oxygen species, SOD1, superoxide dismutase 1

## Abstract

MN (motor neuron) death in amyotrophic lateral sclerosis may be mediated by glutamatergic excitotoxicity. Previously, our group showed that the microdialysis perfusion of AMPA (α-amino-3-hydroxy-5-methyl-4-isoxazole propionate) in the rat lumbar spinal cord induced MN death and permanent paralysis within 12 h after the experiment. Here, we studied the involvement of energy metabolic deficiencies and of oxidative stress in this MN degeneration, by testing the neuroprotective effect of various energy metabolic substrates and antioxidants. Pyruvate, lactate, β-hydroxybutyrate, α-ketobutyrate and creatine reduced MN loss by 50–65%, preserved motor function and completely prevented the paralysis. Ascorbate, glutathione and glutathione ethyl ester weakly protected against motor deficits and reduced MN death by only 30–40%. Reactive oxygen species formation and 3-nitrotyrosine immunoreactivity were studied 1.5–2 h after AMPA perfusion, during the initial MN degenerating process, and no changes were observed. We conclude that mitochondrial energy deficiency plays a crucial role in this excitotoxic spinal MN degeneration, whereas oxidative stress seems a less relevant mechanism. Interestingly, we observed a clear correlation between the alterations of motor function and the number of damaged MNs, suggesting that there is a threshold of about 50% in the number of healthy MNs necessary to preserve motor function.

## Abstract 2

Mitochondrial energy substrates protect against *in vivo* excitotoxic spinal motor neuron degeneration and the consequent paralysis, whereas antioxidants are less efficient. These results allowed to establish a minimal threshold number of spinal motor neurons necessary to preserve motor function.

## INTRODUCTION

The neurodegenerative disease ALS (amyotrophic lateral sclerosis) is characterized by the selective and progressive degeneration of lower and upper MNs (motor neurons), leading to a progressive paralysis and finally death due to respiratory failure, usually 2–5 years after the symptoms onset. The cause of ALS is unknown and the several hypotheses that have been postulated to explain the selective MN death include glutamate-mediated excitotoxicity, mitochondrial dysfunction and oxidative stress (Tovar-y-Romo et al., 2009; Robberecht and Philips, 2013).

Excitotoxicity induced by overactivation of glutamate receptors may be involved in mechanisms of neurodegeneration in ALS, mainly because spinal MNs are particularly vulnerable to overactivation of the Ca^2+^-permeable AMPA (α-amino-3-hydroxy-5-methylisoxazole-4-propionate)-type receptors, both in cultures (Van Den Bosch et al., 2000; Van Damme et al., 2002) and *in vivo* (Corona and Tapia, 2007; Corona et al., 2007). An increase in intracellular Ca^2+^ entering through these receptors results in neuronal death due to activation of lytic enzymes, mitochondrial damage linked to energy metabolism disruption and generation of toxic ROS (reactive oxygen species) (Shaw, 1999; Arundine and Tymianski, 2003; Grosskreutz et al., 2010). Particularly, mitochondrial bioenergetic status is determinant for neuronal survival or death, and alterations in mitochondrial function occur after experimental excitotoxicity and in ALS (Nicholls and Budd, 2000; Duffy et al., 2011; Cozzolino and Carrì, 2012; Santa-Cruz et al., 2012).

Previously, our group developed an *in vivo* model of MN death by means of microdialysis perfusion of AMPA in the rat lumbar spinal cord, which produced a remarkable progressive loss of spinal MNs, leading to permanent paralysis of the ipsilateral hindlimb (Corona and Tapia, 2004, 2008). In this model we found that pyruvate significantly protected against MN loss and paralysis (Corona and Tapia, 2007), probably acting as a supplemental energy substrate restoring mitochondrial function. However, abundant evidence supports the involvement of oxidative stress in ALS pathogenesis as well (Barber and Shaw, 2010; Santa-Cruz et al., 2012), so that the neuroprotective effect of pyruvate may be due also to its antioxidant properties (Desagher et al., 1997; Kim et al., 2005; Wang et al., 2007). Therefore the aim of the present work was to clarify the role of energy metabolic deficiencies and oxidative stress in the AMPA-induced spinal MN death *in vivo*. For this purpose, we assessed the neuroprotective effects of the energy substrates lactate and creatine, of α-ketobutyrate and β-hydroxybutyrate, which can supply energy and also have antioxidant properties, and of ascorbate, glutathione and glutathione ethyl ester, which are solely antioxidants. In addition, we determined the effect of AMPA on protein oxidation and ROS formation.

## MATERIALS AND METHODS

### Animals

Adult male Wistar rats weighing 280–310 g were used in all the experiments and handled in accordance with the Rules for Research in Health Matters (Mexico), with approval of the local Animal Care Committee. All efforts were made to minimize suffering of the animals. Animals were housed with a 12 h light/dark cycle and with food and water *ad libitum*.

### Microdialysis procedure and drug administration

Microdialysis in the lumbar spinal cord was carried out essentially as previously described (Corona and Tapia, 2004, 2007), including the collection of perfused media fractions for the measurement of amino acids. Briefly, rats were anesthetized with 5% halothane or isoflurane in a 95% O_2_/5% CO_2_ mixture, placed in a stereotaxic spinal unit (Kopf), and maintained under low anesthesia (~0.8–1% halothane or isoflurane) during surgery. A ~1–2 mm diameter hole was drilled at the second–third lumbar laminae and a microdialysis probe (1 mm long and 0.24 mm diameter, CMA/7, Solna) was slowly lowered down into the right dorsal horn of the spinal cord. A Krebs–Ringer medium [118 mM NaCl, 1.2 mM KH_2_PO_4_, 4.7 mM KCl, 1.18 mM MgSO_4_, 25 mM NaHCO_3_, 10 mM glucose and 2.5 mM CaCl_2_ (pH 7.4)], was continuously perfused at a flux rate of 2 μl/min, using a microsyringe mounted on a microinjection pump (CMA/100, Solna), during 160 min, as follows: 60 min equilibration period, followed by 37.5 min during which three 12.5 min fractions were collected for the measurement of the basal concentration of amino acids; then AMPA-containing medium (6 mM, Tocris) was perfused during 25 min, with collection of two 12.5 min fractions, and finally medium was perfused during 37.5 min, with collection of three 12.5 min recovery fractions. The energy substrates and antioxidants tested were always present during the 160 min of the microdialysis procedure, except in the controls perfused only with Krebs medium and in the rats perfused only with AMPA. Sodium pyruvate (20 mM), L-(+)-lactic acid (20, 50 and 100 mM), creatine monohydrate (20 mM), DL-β-hydroxybutyric acid sodium salt (20 and 50 mM), α-ketobutyric acid (20 mM), L-ascorbic acid (10, 20 and 50 mM), reduced L-glutathione (20 and 50 mM), reduced glutathione ethyl ester (20 and 50 mM), or the latter combined with sodium pyruvate (20 mM each) were dissolved in the Krebs–Ringer perfusion medium, adjusting the pH to 7.4 if necessary. All compounds were obtained from Sigma–Aldrich. Osmolarity was maintained by reducing the NaCl concentration proportionally. Initial concentrations of the compounds tested were chosen on the basis of previous results from this laboratory for pyruvate. At the end of the microdialysis procedure, the skin was sutured and anesthesia was discontinued. Rats were kept in individual cages with water and food *ad libitum*, and observed periodically during the next 24 h. At this time they were fixed for the histological analysis, as described below.

Glutamate and aspartate concentrations in the 25 μl dialysate fractions collected along the microdialysis procedure were measured by HPLC as previously described (Massieu et al., 1995; Corona and Tapia, 2004). No significant changes in the levels of these amino acids were observed under any of the experimental conditions used, and therefore no data are shown on amino acid quantification.

### Assessment of motor function

Besides the observation of walking and general motor behavior, motor performance was evaluated 6 and 24 h after the experiment by the rotarod test (Columbus Instruments), as previously described (Corona and Tapia, 2007).

### Histology and ChAT (choline acetyltransferase) immunohistochemistry

At 24 h after surgery, subsequent to the assessment in the rotarod test, the animals were fixed for histological and ChAT immunohistochemical analyses, exactly as previously described (Corona and Tapia, 2007). Briefly, transverse sections (50 μm thick) of the spinal cord segment where the cannula was introduced were obtained in a cryostat, and alternate sections were stained with Cresyl Violet or immunostained for ChAT, so that histological and immunohistochemical changes can be correlated. The morphologically undamaged MNs (i.e. large neurons, with a soma diameter >20 μm and a distinguishable nucleus, similar in appearance to those of the contralateral ventral horn and to those in control rats) were counted in a 10× microscopic field. Five to seven sections, 50 μm apart, where the trace of the cannula was evident, were counted in the ipsilateral and contralateral ventral horn of each rat, and the values were averaged. For ChAT immunohistochemistry, goat polyclonal anti-ChAT antibody (1:200; Chemicon), biotinylated-conjugated horse anti-goat IgG (1:200; Vector) and avidin-Texas Red conjugate (1:200, pH 8.2; Vector) were used. Sections were mounted on silane (Sigma)-covered slides and coverslipped with fluorescent mounting medium (DAKO); they were examined in a Nikon microscope equipped with an epifluorescence attachment. Parallel sections processed in the absence of the primary antibody showed no immunostaining.

### 3-NT (3-nitrotyrosine) and ChAT double immunohistochemistry

3-NT was assessed in order to study the effect on protein oxidation of AMPA, administered by microdialysis at a 6 mM concentration, as described above. Because MN loss started ~3–6 h after AMPA perfusion (Corona and Tapia, 2008), in these experiments rats were transcardially fixed 2 h after as described above, so that oxidation could be measured at the beginning of the degeneration process rather than when MNs were already damaged. After fixation, spinal cords were post-fixed, transferred to sucrose and transverse sections 50 μm thick were obtained in a cryostat. For a positive control, nitration of tyrosine was carried out directly in spinal cord sections of intact rats by means of peroxynitrite generation *in situ*, accomplished by mixing 0.1 M sodium nitrite with 0.1 M hydrogen peroxide in acetate buffer, pH 5, for ~20 min at room temperature (Viera et al., 1999).

Free-floating sections were blocked with 5% BSA in PBS-Triton X-100 (0.3%) for 2 h, and then incubated with goat polyclonal anti-ChAT (1:200) and mouse monoclonal anti-nitrotyrosine antibodies (1:50; Sigma–Aldrich), for 7 days at 4°C. Sections were washed three times for 10 min in PBS-Triton X-100 and incubated with biotinylated-conjugated horse anti-goat IgG (1:200) for 1–2 h. After three washes, sections were incubated for 2 h with avidin–Texas Red conjugate (1:200, pH 8.2), washed three times, and incubated with FITC-goat anti-mouse IgG (1:200; Zymed) for 2 h. Finally, sections were washed and mounted on silane-covered slides and coverslipped with fluorescent mounting medium. Sections were visualized under confocal microscopy (Olympus IX81). Merged images are the overlay of two laser sections in the Z plane, using the Olympus Fluoview laser scanning FV1000 Ver. 3.0 Viewer.

### Measurement of ROS in spinal cord homogenates

The effect of 6 mM AMPA perfusion on ROS production was also studied at ~2 h after the experiment, for the reasons mentioned above. In these experiments the microdialysis procedure was carried out as described above but bilaterally, using one microdialysis probe on each dorsal horn. The two ventral horns of the perfused lumbar segments were dissected out on ice and homogenized together in 1200 μl of buffer containing 220 mM mannitol, 70 mM sucrose, 2 mM MOPS and 1 mM EGTA (pH 7.4). ROS production was measured by the oxidation of 2′,7′-dichlorodihydrofluorescein (Halliwell and Whiteman, 2004). Aliquots (10 μl) of the homogenates were mixed in triplicate with 190 μl of 10 μM 2′,7′-dichlorodihydrofluorescein diacetate (Sigma) in the same medium with the addition of 2 mM phosphate, 20 mM KCl and 1 mM MgCl_2_, and incubated at 37°C. Fluorescence signals (488 nm excitation and 525 nm emission wavelength respectively) were recorded every minute for 60 min in a Synergy HT Multi-Mode Microplate Reader (BioTek). The biuret method was used for the determination of protein concentration in the homogenates.

### Statistical analysis

Comparisons regarding rotarod scores, number of MNs and ROS production were carried out using ANOVA followed by a Tukey's *post hoc* test. A value of *P*<0.05 was considered statistically significant.

## RESULTS

**Figure 1:**
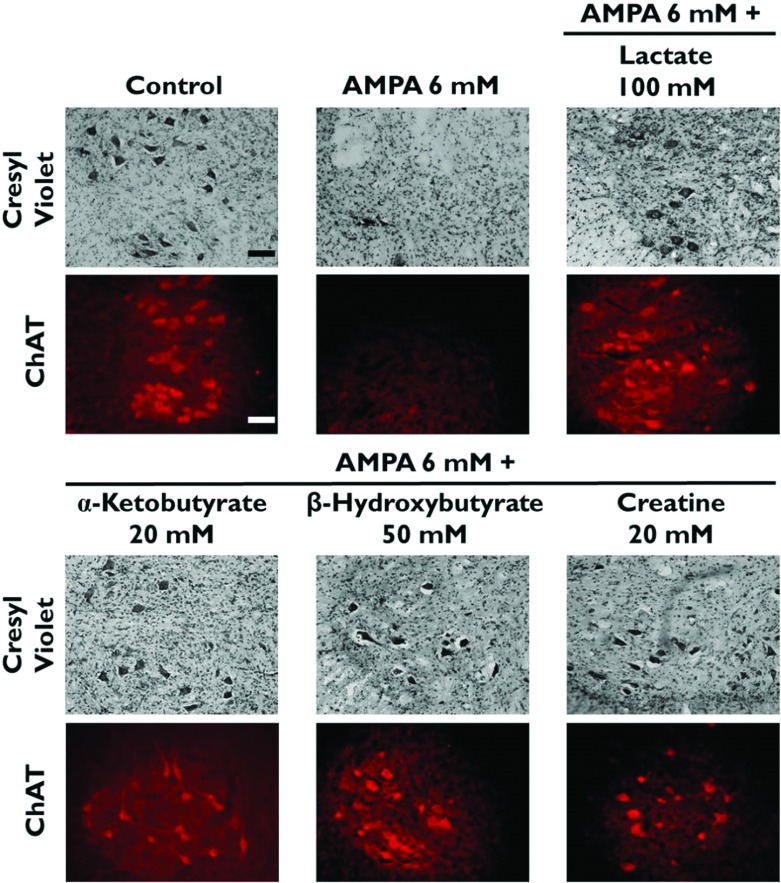
Energy substrates protect MNs from AMPA-induced degeneration Cresyl Violet and ChAT immunocytochemistry representative micrographs of the ipsilateral ventral horn of rats treated as indicated, 24 h after the experiment. Note the severe MN loss caused by AMPA and the protective action of the energy substrates. No neuronal damage was observed in the contralateral ventral horn in any case. Scale bars=100 μm. See Figure 4 for quantitative data.

**Figure 2:**
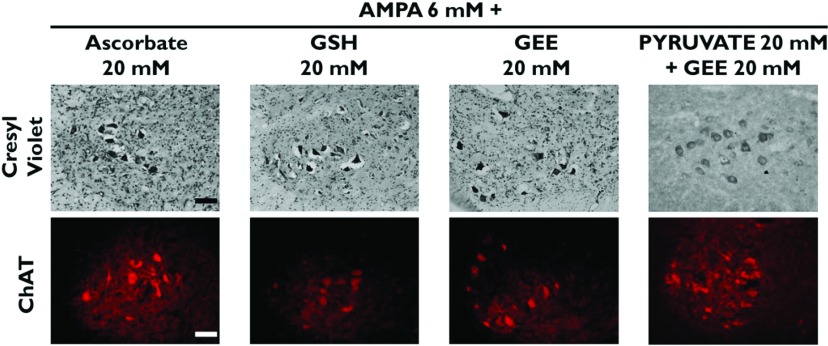
Antioxidants partially protect MNs from AMPA-induced degeneration Cresyl Violet and ChAT immunocytochemistry representative micrographs of the ipsilateral ventral horn of rats treated as indicated, 24 h after the experiment. Note that co-perfusion of an antioxidant and an energy substrate does not have a synergic effect. Compare with control and with AMPA alone in Figure 1. Scale bars=100 μm. See Figure 4 for quantitative data.

**Figure 3:**
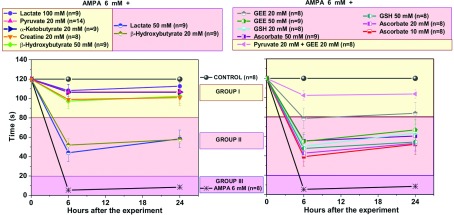
Rotarod performance of rats treated with AMPA and AMPA+energy substrates or antioxidants Time remaining in the rotarod, with a cut-off of 120 s, 6 and 24 h after the experiment. Control rats (perfused with Krebs–Ringer medium) did not fall down before the cut-off time. Results are shown divided into three groups according to the animals performance: rats treated with AMPA alone (group III, purple background) presented a complete and permanent paralysis of the ipsilateral hind limb and fell down in less than 20 s, 6 h after the experiment. Group I (yellow background in graph and list) includes rats treated with AMPA+the compounds that at the concentrations indicated exerted remarkable protection, since none of these rats showed any sign of paralysis or other motor deficit and remained in the rotarod for at least 90 s. Group II (pink background in graph and list) includes rats treated with AMPA+the compounds that at the concentrations indicated exerted partial protection, because these animals showed partial paralysis and fell from the rotarod between 40 s and 70 s. Note that only energy substrates are in group I whereas all antioxidants are in group II. Data are means±S.E.M. for the number of rats indicated in parentheses. All values of groups I and II differed significantly from group III (*P*<0.001). Differences between control and group I values were not significant (*P*>0.05), except in the case of creatine, β-hydroxybutyrate (50 mM), and pyruvate+GEE (*P*<0.05); differences were significant between groups I and II (*P*<0.05, except in the case of 20 mM GEE, in which four rats behaved as those of group I and four rats as those of group II).

**Figure 4:**
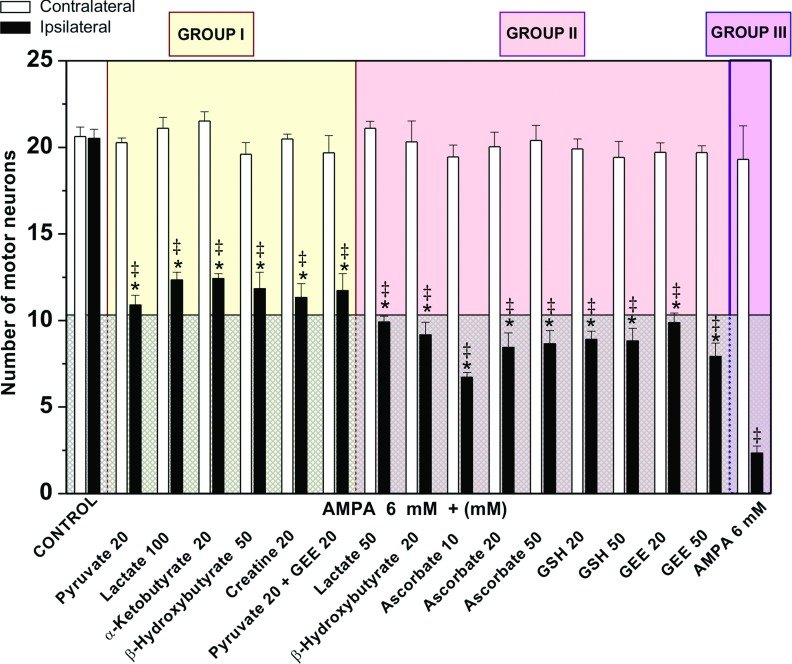
Number of healthy MNs in control rats and rats treated with AMPA and AMPA+substrates and antioxidants Neurons of the ipsilateral and contralateral ventral horns were counted 24 h after the experiment and are classified in the three different groups described in Figure 3, using the same background colors. The horizontal line that divides the graph indicates 50% of the number of healthy MNs as compared with control rats. Note the remarkable (89%) loss of MNs in the ipsilateral horn in the AMPA group III, and that all compounds of group I protected by more than 50% whereas the protection by all compounds of group II was below 50% (shaded area). Data are means±S.E.M. for the number of rats indicated in parentheses in Figure 3 for each group. *P<0.001 vs group III. ‡*P*<0.001 vs the corresponding contralateral ventral horn. All group II values, except lactate, β-hydroxybutyrate and 20 mM GEE, differed significantly from group I values (*P*<0.05). Figure 6 shows a correlation between the data of this Figure and those of Figure 3.

Figures 1 and 2 show representative micrographs of ventral horns of rats showing the clearest effects of the compounds tested on MN preservation, and Figures 3 and 4 show quantitative data of the results of the rotarod test and of MN counts respectively, grouped according to the effectiveness of the protective action, as explained in the Discussion.

### Energy substrates prevent AMPA-induced paralysis and neurodegeneration

Control rats perfused only with the substrates or the antioxidants dissolved in the Krebs–Ringer medium did not show any behavioral alteration at any time. No histological alterations, other than the mechanical damage caused by the probe in the dorsal spinal horn, were observed in these control animals, 24 h after the experiment (*n*≥3).

We confirmed that 6 mM AMPA produces a dramatic MN loss (~90%) in the ipsilateral ventral horn and consequently a total paralysis of the ipsilateral hindlimb 24 h after the experiment (Corona and Tapia, 2004, 2008). We also confirmed (Corona and Tapia, 2007), that 20 mM pyruvate exerts a remarkable neuroprotection, preventing paralysis and diminishing MN loss by >50% (Figures 1, 3 and 4). In contrast, when we tested 20 mM lactate no protective effect at all was observed (results not shown). However, 50 mM lactate partially prevented the motor impairment, assessed by the rotarod test (Figure 3), and MN loss diminished to ~52% (Figure 4). This protection was notably enhanced when lactate concentration was increased to 100 mM, since no paralysis was observed, the rotarod performance was only slightly affected (Figure 3) and MN loss was only ~40% (Figures 1 and 4). Notably, the protective effect of 100 mM lactate was equivalent to that of 20 mM pyruvate.

Creatine and α-ketobutyrate, both at a 20 mM concentration, also exerted a remarkable protection against AMPA-induced excitotoxicity, which was very similar to that of 100 mM lactate and of 20 mM pyruvate, in terms of motor behavior, rotarod performance and MN death (Figures 1, 3 and 4). The ketone body β-hydroxybutyrate was tested also at 20 and 50 mM concentrations, and its protective effect was similar to that of 50 and 100 mM lactate respectively, since the animals showed comparable rotarod performance and MN loss (Figures 1, 3 and 4).

### Antioxidants only partially prevent AMPA-induced motor dysfunction and MN loss

Ascorbate was tested at 10, 20 and 50 mM concentrations. Although a significant protection against AMPA was observed, in both rotarod test and MN loss the protection was similar with all concentrations and was also similar to that exerted by 50 mM lactate (Figures 2, 3 and 4).

GSH, one of the major antioxidant defenses of the cell, was tested at 20 and 50 mM concentrations and, as shown in Figures 2, 3 and 4, the protection observed was strikingly similar to that produced by 20 and 50 mM ascorbate, in terms of both rotarod performance and MN loss. Since it has been reported that intracellular GSH levels are elevated more efficiently after the administration of GEE (GSH ethyl ester) than after GSH administration (Zeevalk et al., 2007), we also tested GEE as a protector against AMPA. Although the results with 20 mM GEE were more variable than with GSH (some rats behaved better than others in the rotarod test and showed >50% MN protection), the effect exerted by both 20 mM and 50 mM concentrations did not differ significantly from that by GSH (Figures 2, 3 and 4).

Since in none of the above experimental conditions the protection of the healthy MNs exceded 65–70%, we tested whether the combined perfusion of an energy substrate and an antioxidant resulted in a better protection. We chose 20 mM pyruvate+20 mM GEE and the results obtained, both in the rotarod performance and MN number, were very similar to those with pyruvate alone, indicating that the effects were not additive (Figures 2, 3 and 4).

### 3-NT and ROS production were not affected by AMPA during the initial stages of the MN degeneration process

To further study the involvement of oxidative stress in the MN degenerating process, the oxidative stress markers 3-NT and ROS were measured. These determinations were carried out at 1.5–2 h after the microdialysis procedure, because we have observed previously that the MN degenerating process starts rapidly after AMPA perfusion and is observed as early as 3 h after AMPA microdialysis (Corona and Tapia, 2008).

**Figure 5:**
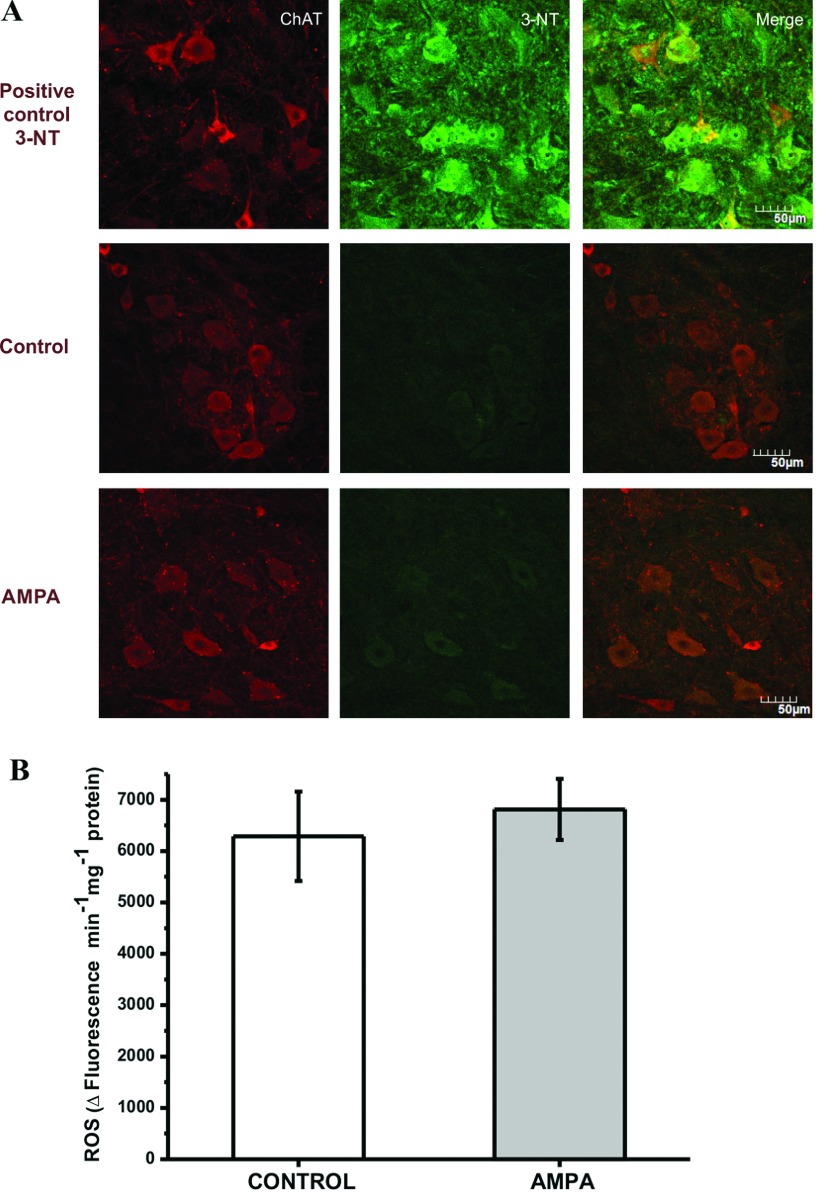
Double immunohistochemistry for 3-NT and ChAT (A), and ROS production rate (B), during the first stages of the MN degeneration process (**A**) Representative micrographs ~2 h after the microdialysis procedure (*n*=8). A positive control for 3-NT detection, as described in the Materials and Methods, is shown at the top line. Only a faint 3-NT labeling was detected in both control and AMPA-treated rats. (**B**) ROS production in the ventral horns is not affected by AMPA, ~2 h after the microdialysis procedure. Means±S.E.M. (*n*=8).

As shown in Figure 5(A), although positive controls verified the detection of 3-NT in the spinal cord sections, in both control and AMPA-treated rats we observed only a very tenuous staining for 3-NT, which could not be related to the ChAT-labeled MNs. As shown in Figure 5(B), there was no difference in the ROS production rate in ventral horn homogenates between control and AMPA-perfused rats.

## DISCUSSION

The study of the neuroprotective effects of various compounds with different properties can be useful to find out and understand which mechanisms are involved in selective degeneration of spinal MNs. We have used this approach to study the participation of mitochondrial energy deficiency and oxidative stress in the rapid neuronal death produced by AMPA receptor overactivation.

Of all compounds tested as protectors, pyruvate was one of the most effective since, as previously described (Corona and Tapia, 2007), at a 20 mM concentration it reduced MN death and completely prevented paralysis and motor impairment. In neurons, pyruvate is the most important mitochondrial substrate derived from glycolysis and, as discussed below, can also act as an antioxidant. When its protection was compared with that of lactate, an energy substrate that is not an antioxidant *per se*, we found a dose-dependent neuroprotection, but a 100 mM concentration was required to protect as compared with 20 mM pyruvate, suggesting that lactate is oxidized to pyruvate inside neurons by lactate dehydrogenase. Both lactate and pyruvate cross the blood–brain barrier, are transported to the glial cells and neurons by the monocarboxylate transporters, and can be used as energy substrates through the TCA (tricarboxylic acid) cycle (Nicholls and Budd, 2000; Smith et al., 2003; Aubert et al., 2005; Bouzier-Sore et al., 2006; O’Brien et al., 2007). In fact, astrocytes can protect neurons *in vitro* through the release of lactate, so that neighboring neurons can use it as an energy substrate, and this facilitates neuron recovery after a bioenergetic insult (Izumi et al., 1997; Schurr et al., 1997). That lactate is used as a significant source of fuel for brain metabolism, predominantly in neurons, has been shown also *in vivo* under different energy requirement conditions, including lactate released from astrocytes or transported from blood (reviewed by Pellerin et al., 2007; Figley, 2011). Furthermore, it has been recently shown that the disruption of monocarboxylate transporter 1 present in oligodendroglia produces neuronal and axonal damage and that its expression is reduced in affected regions of ALS patients and mutant SOD1 (superoxide dismutase 1) mice (Lee et al., 2012).

However, neuroprotective effects of pyruvate have also been associated with its ability to exert antioxidant effects. Pyruvate and other α-ketoacids, including α-ketobutyrate, protected cultured striatal neurons against toxicity induced by H_2_O_2_, whereas lactate did not protect; this was attributed to the ability of α-ketoacids to react with H_2_O_2_ in a decarboxylating reaction (Desagher et al., 1997). Pyruvate protects also human neuroblastoma cells by means of its antioxidant actions in the mitochondria, since it suppressed mitochondrial superoxide production, mitigated mitochondrial transmembrane potential collapse induced by oxidative stress, and attenuated H_2_O_2_-induced ROS formation and cell death (Wang et al., 2007). When we tested α-ketobutyrate, another α-ketoacid that can react with H_2_O_2_ but can also be oxidized by α-ketobutyrate dehydrogenase, we observed a protective effect similar to that of pyruvate at equivalent (20 mM) concentrations, suggesting that they act by similar mechanisms.

It is well known that ketone bodies can be used as energy substrates in the CNS under some circumstances and they can exert protective effects in various pathological conditions (Maalouf et al., 2009). We tested the neuroprotective effect of β-hydroxybutyrate, a ketone body that can be oxidized to acetoacetate, which gives rise to two molecules of acetyl-CoA and therefore can promote mitochondrial energy production. β-Hydroxybutyrate exerted a dose-dependent protection, lessening motor impairment and MN loss. In agreement with these results, SOD1-G93A transgenic mice fed with a ketogenic diet preserved motor function longer, body weight loss was decreased and spinal MN loss was diminished; in addition, in these animals mitochondrial function assessed as ATP synthesis was increased, and *in vitro* β-hydroxybutyrate stimulated ATP synthesis and prevented MN loss induced by rotenone (Zhao et al., 2006). Further, treatment of SOD1-G93A mice with caprylic triglyderide (a medium-chain triglyceride that is metabolized into ketone bodies) attenuated progression of symptoms and promoted mitochondrial oxygen consumption rate measured *ex vivo* (Zhao et al., 2012).

We observed a good neuroprotective effect of 20 mM creatine, which helps to provide energy by stimulating ATP formation from the phosphocreatine reserve when an unexpected energy demand depletes ATP, which is what most likely occurs during AMPA-induced excitotoxicity. This neuroprotection is in agreement with results obtained in SOD1-G93A mice, showing that oral administration of creatine improved motor performance, extended survival and prevented MN loss (Klivenyi et al., 1999). However, at least two human clinical trials testing creatine in ALS patients showed no benefical effects (Groeneveld et al., 2003; Shefner et al., 2004). Ascorbate is a cofactor in many enzymatic reactions and a potent antioxidant. In the CNS its concentration is much higher than in plasma, suggesting that this compound plays a crucial role in ROS removal (Rice, 2000). Neuroprotective effects of ascorbate against damage induced by excitatory amino acids and ischemia have been shown *in vitro* and *in vivo* (Majewska and Bell, 1990; MacGregor et al., 1996; Stamford et al., 1999; Kim et al., 2008), and SOD1-G93A mice fed with a diet supplemented with ascorbate survived longer, but symptom onset was not delayed (Nagano et al., 2003). In our experiments we observed only partial protection with all of the concentrations tested, not better than 50 mM lactate.

When we tested GSH, the major antioxidant defense in the cell, 20 and 50 mM concentrations exerted only partial protection, similar to ascorbate. There is little information characterizing the cellular uptake of GSH, but since one study showed that administration of GEE can increase intracellular GSH concentration and provides neuroprotection against oxidative stress or chronic mitochondrial damage (Zeevalk et al., 2007), we tested this ester. The results were somewhat contradictory, since 50 mM GEE exerted less protection than 20 mM and in the latter group half of the rats were highly protected and reduced MN loss by more than 50%, whereas the rest of the rats were only partially protected. GSH plays an important role in detoxification of ROS in brain, reacting directly with superoxide, nitric oxide and its derivatives or the hydroxyl radical in non-enzymatic reactions, or indirectly by providing reducing capacity for several GSH-dependent enzymes (Dringen, 2000). Furthermore, GSH can be translocated to mitochondria where it contributes to defense against ROS-mediated damage, and against reactive nitrogen species (Heales and Bolaños, 2002). So, the results obtained with GSH and GEE are relevant and could be due to other neuroprotective antioxidants that are reduced by GSH, like vitamin E (Forman et al., 2009).

We then tested the hypothesis that neuroprotective properties of pyruvate as an energy substrate and GEE as an antioxidant would be additive, but the results show that there is no synergy effect, since the number of healthy MNs was not significantly higher than with pyruvate alone. This suggests that the main protection is due to an energy mechanism, whereas oxidative stress is perhaps a secondary event resulting from mitochondrial dysfunction. The findings that AMPA did not increase 3-NT staining and ROS production rate, at a time when, according to our previous findings (Corona and Tapia, 2008), MNs are in the initial stages of the degeneration progress, agree with this conclusion, although the participation of ^•^NO-dependent oxidative stress in later stages of the degeneration cannot be excluded. So, the augmented reactive oxygen and nitrogen species could directly damage mitochondria as well, aggravating mitochondrial dysfunction and creating a vicious circle that ends up in MN death.

### There is a threshold in the number of MNs necessary to prevent motor impairment and paralysis

**Figure 6:**
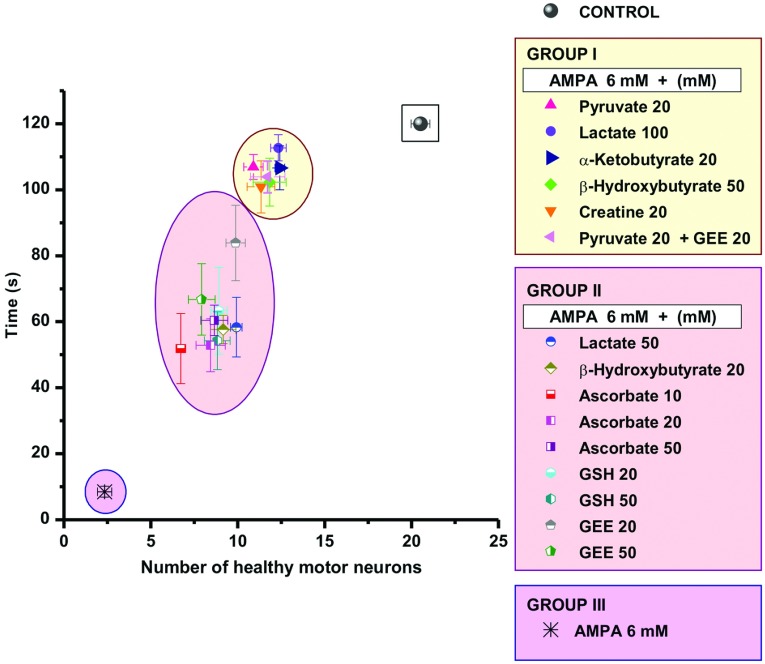
Correlation between the number of healthy MNs and the rotarod performance of the animals This graph shows the number of healthy MNs in the ipsilateral ventral horns of the spinal cords of rats in each experimental condition tested plotted vs the time animals remained in the rotarod 24 h after the experiment. Plotted data, grouping of data and colors in the graph and the list are as described in Figures 3 and 4.

We analyzed the relationship between the motor performance of the rats in the rotarod and the number of healthy MNs under all experimental conditions used and those of previous works in this acute model of excitotoxic MN degeneration (Corona and Tapia, 2004, 2007, 2008). As shown in Figures 3, 4 and 6, this correlation clearly shows, first, that a small difference in the number of MNs results in a remarkable difference in motor function, and second, that there is a threshold in the percentage of healthy MNs required to completely prevent motor function impairment. In fact, in all cases as long as more than 50% of MNs were preserved, paralysis and motor dysfunction were completely prevented (group I, yellow background in Figures 3, 4 and 6), whereas when MN preservation is slightly below this threshold value motor deficiencies leading to rotarod falling within 60–80 s occur (group II, pink background). A severe MN loss produces total and permanent paralysis of the ipsilateral hind limb and falling from the rotarod in a few seconds (group III, purple background). Figure 6 summarizes the data supporting this threshold hypothesis and the correlation between the number of healthy MNs and motor performance. It is noteworthy that besides these results, the findings with other compounds that have shown protective action in this acute model of excitotoxic MN degeneration also suggest that a ~50% MN loss correlates with motor deficits and none of them have protected 100%. The other compounds tested are the AMPA receptor antagonist 2,3-dihydroxy-6-nitro-7-sulfamoyl-benzo(F)quinoxaline (Corona and Tapia, 2004); the selective blocker of the Ca^2+^-permeable AMPA receptors 1-naphthyl acetyl spermine and the intracellular Ca^2+^ chelator 1,2-bis(2-aminophenoxy)ethane-N,N,N',N'-tetraacetic acid tetrakis (acetoxymethyl ester) (Corona and Tapia, 2007); and the calpain inhibitor leupeptin (Corona and Tapia, 2008).

It is noteworthy that all rats treated with antioxidants at any concentration tested belong to the partially protected group II, whereas all the well-protected rats of group I were treated with energy substrates. This supports our previous conclusion that oxidative stress is contributing, but is not the main triggering event, of AMPA-induced MN degeneration, whereas mitochondrial energy metabolism deficit seems crucial in the process of neurodegeneration.

As mentioned in the Introduction, the mechanism by which AMPA-induced excitotoxicity may generate mitochondrial energy dysfunction is probably mediated by an increase in intracellular Ca^2+^ due to the overactivation of Ca^2+^-permeable AMPA receptors (Corona and Tapia, 2007). In fact, the maintenance of intracellular calcium homoeostasis, whether extruding it from the cell or sequestering it in intracellular stores, require high-energy expenditure, and on the other hand, mitochondrial damage related to Ca^2+^ homoeostasis alterations is involved in experimental MN degeneration, and in ALS (Carriedo et al., 2000; Nicholls and Budd, 2000; Grosskreutz et al., 2010; Santa-Cruz et al., 2012).

In conclusion, the mechanisms involved in *in vivo* excitotoxic spinal MN degeneration seem to be multifactorial, since none of the compounds tested in our model has completely prevented MN death. However, our results suggest that mitochondrial energy deficits are crucially involved in this degeneration, whereas oxidative stress seems a less relevant mechanism. Although it is not certain that excitotoxicity is the first cause of MN degeneration in ALS, it is very likely that it participates in neuronal death in this disease due to the selective vulnerability of MNs to AMPA, so these mechanisms could be involved in ALS MN degeneration. With the limitation that our experimental model is restricted to two or three lumbar segments of the spinal cord, we consider that the correlation showing that there seems to be a minimal threshold number of spinal MNs necessary to preserve hind limb movements is of interest regarding the analysis of the progress of ALS in humans and the potential therapeutic strategies for this devastating disease.
